# A Novel Method Based on Hydrodynamic Cavitation for Improving Nitric Oxide Removal Performance of NaClO_2_

**DOI:** 10.3390/ijerph20043684

**Published:** 2023-02-19

**Authors:** Liguo Song, Yuhang Wei, Chengqi Deng, Jingang Yang, Hao Sui, Feng Guo, Lingrun Meng, Xingda Zhao, Shiping Wei, Deping Sun, Zhitao Han, Minyi Xu, Xinxiang Pan

**Affiliations:** 1 Marine Engineering College, Dalian Maritime University, Dalian 116026, China; 2 Liaoning Research Center for Marine Internal Combustion Engine Energy-Saving, Dalian 116026, China; 3 School of Electronics and Information Technology, Guangdong Ocean University, Zhanjiang 524088, China

**Keywords:** hydrodynamic cavitation, NaClO_2_, wet removal of NO, ship, exhaust gas treatment

## Abstract

In the removal of nitric oxide (NO) by sodium chlorite (NaClO_2_), the NaClO_2_ concentration is usually increased, and an alkaline absorbent is added to improve the NO removal efficiency. However, this increases the cost of denitrification. This study is the first to use hydrodynamic cavitation (HC) combined with NaClO_2_ for wet denitrification. Under optimal experimental conditions, when 3.0 L of NaClO_2_ with a concentration of 1.00 mmol/L was used to treat NO (concentration: 1000 ppmv and flow rate: 1.0 L/min), 100% of nitrogen oxides (NO_x_) could be removed in 8.22 min. Furthermore, the NO removal efficiency remained at 100% over the next 6.92 min. Furthermore, the formation of ClO_2_ by NaClO_2_ is affected by pH. The initial NO_x_ removal efficiency was 84.8–54.8% for initial pH = 4.00–7.00. The initial NO_x_ removal efficiency increases as the initial pH decreases. When the initial pH was 3.50, the initial NO_x_ removal efficiency reached 100% under the synergistic effect of HC. Therefore, this method enhances the oxidation capacity of NaClO_2_ through HC, realizes high-efficiency denitrification with low NaClO_2_ concentration (1.00 mmol/L), and has better practicability for the treatment of NO_x_ from ships.

## 1. Introduction

Over 80% of global trade transport is through ships [1]. Furthermore, it is estimated that the average annual growth rate of international maritime transport trade will be 3.5% from 2019 to 2024 [2]. However, there are increasing concerns about the environmental problems caused by the ships’ exhaust. The ships’ exhaust mainly contains particulate matter (PM), nitrogen oxides (NO_x_), carbon dioxide (CO_2_), sulfur oxides (SO_x_), and other substances hazardous to human health and the environment [3,4,5,6]. Additionally, NO_x_ is the most difficult to remove [7,8]. Selective catalytic reduction (SCR) and exhaust gas recirculation (EGR) can remove NO from ship exhaust. SCR uses catalysts and ammonia to reduce NO_x_ emissions. However, the removal of NO by SCR is greatly affected by temperature [9], and SO_x_ and water in the exhaust gas from ships can cause catalyst poisoning [2]. EGR can reduce NO production at the source. However, increasing the EGR rate will lead to incomplete combustion, causing an increase in the PM [10] and reducing fuel economy [11]. SCR and EGR can only deal with NO, not SO_2_ and PM simultaneously. Wet scrubbing technology has the advantage of treating multiple pollutants simultaneously and has attracted extensive attention from scholars [12]. The NO_x_ emissions from ships consist of 90–95% NO. Moreover, NO is difficult to dissolve in water [13]. In wet removal of NO, it is oxidized with oxidants, and then NO_x_ removal is promoted with absorbents [14]. High-potential oxidants include H_2_O_2_ [15], Fenton-like reagents [16,17], persulfate salts [18,19], NaClO_2_ [20], and KMnO_4_ [21]. The absorbents include sodium humate (HA-Na) [22,23], NaSO_3_ [24], and Ca(OH)_2_ [25]. Hao et al. [26] compared the performance of different advanced oxidation processes (AOPs) such as UV/H_2_O_2_, UV/NaClO, UV/Na_2_S_2_O_8_, and UV/NaClO_2_ in terms of NO removal. UV/NaClO_2_ has the best NO conversion performance among these AOPs. Additionally, secondary pollution to the ocean can be avoided when NaClO_2_ is used as the oxidant, since it can be converted into sodium and chloride ions [27]. Scholars have studied the removal of NO by NaClO_2_ using different reactors. Deshwal et al. [28] used a bubble reactor to remove NO. When the concentration of NaClO_2_ was 200.00 mmol/L, the NO_x_ removal efficiency reached 81%. However, the bubble reactor has problems such as increasing the exhaust back pressure of the diesel engine and poor gas–liquid mass transfer. Han et al. [29] used a cyclic scrubbing system containing a fine-droplet spray nozzle to remove NO. When the concentration of NaClO_2_ is 10.00 mmol/L, the NO_x_ removal efficiency could reach 87.7%. When dealing with actual ship flue gas, particulates may tend to clog the nozzles, and their operation requires high energy consumption. Hao et al. [30] used an vaporization device to evaporate the composite oxidant containing NaClO_2_ to pre-oxidize NO and then absorb NO_x_ through an absorption device containing a HA-Na solution. When the concentration of NaClO_2_ is 80.00 mmol, the NO_x_ removal efficiency can reach 92.0%. This two-stage system can effectively remove NO_x_, but the complex device and the use of various drugs increases the cost. The HC reactor has a higher gas–liquid mass transfer coefficient [31], and the special reaction conditions generated by cavitation can also promote the removal of NO_x_. In addition, the HC reactor system is simple and flexible. It can be arranged in groups, making efficient use of the limited space on the ship while simultaneously desulfurizing, denitrifying, and removing PM without clogging. In this study, we combined HC with 1.00 mmol/L NaClO_2_ to remove NO, thereby increasing the NO_x_ removal efficiency and achieving effective denitrification in a single reactor.

HC is widely used in sewage treatment and pretreatment of wood cellulose [32,33], but there are few studies on the use of HC for the denitrification of ship exhaust gas. Song et al. [34] applied the HC reactor in combination with chlorine dioxide (ClO_2_) to remove NO. When the ClO_2_ solution concentration was 1.00 mg/L, NO_x_ removal efficiency exceeded 90% for 100 s, and the outlet NO_2_ concentration was very low (17 ppmv). Yang et al. [35] studied the effects of pressure difference (ΔP) in the HC reactor, solution temperature, NO concentration, gas flow rate, solution pH, and ClO_2_ concentration on denitrification, determined the optimal denitrification conditions, and discussed the reaction pathway. Then, Yang et al. [36] used HC and ClO_2_ to conduct non-circulation desulfurization and denitrification research. When ClO_2_ with a concentration of 600.00 mg/L was added at a rate of 6.67 mL/min, the removal rate of SO_2_ was close to 100%, and the removal rate of NO_x_ was 95.0%. NO_2_ is produced during NO removal by wet oxidation. However, the concentration of NO_2_ generated when HC removes NO is low. Therefore, Song et al. [37] carried out research on the removal of NO_2_ by H_2_O_2_, NaS_2_O_8_, NaClO_2_, and ClO_2_ under HC conditions, clarified the enhanced removal mechanism of HC on NO_2_, and found that NaClO_2_ with too high of a concentration (10.00 mmol/L) was not conducive to the removal of NO_2_. Hydrodynamic cavitation combined with ClO_2_ for NO removal poses the issue of ClO_2_ evaporation. This decreases the drug’s utilization rate and is not conducive to long-term NO removal. By adjusting the solution’s pH and other conditions, it is possible to regulate the rate at which the proper concentration NaClO_2_ produces ClO_2_. This can reduce the escape of ClO_2_ and prolong the time for the solution to remove NO. In addition, the cost of NaClO_2_ is lower than that of ClO_2_, so this paper studied hydrodynamic cavitation combined with NaClO_2_ for denitrification.

This study investigated the effects of differential pressure ∆P, initial pH, reaction temperature, and NaClO_2_ concentration on NO removal under HC. Under optimal experimental conditions, using a 1.00 mmol/L NaClO_2_ solution with 3.0 L volume to remove NO (concentration: 1000 ppmv, flow rate: 1.0 L/min), NO_x_ could be completely removed in 8.22 min. Meanwhile, the duration of 100% NO removal was as long as 15.10 min. Furthermore, this method significantly improved the NO_x_ removal efficiency and reduced the NaClO_2_ concentration compared to previously reported studies [20,22,38,39]. This method provides a novel possibility for the future treatment of ship exhaust gases.

## 2. Experimental

### 2.1. Reagents and Materials

The HC reactor was acquired from Mazzei Injector Company in Bakersfield, CA, USA; the Model 287 Venturi was used in the experiments. The cavitation chamber is constructed from glass-filled polypropylene. Structure and dimensions are depicted in detail in Figure S1 of the Supplementary Materials. This experiment also utilized a flue gas analyzer, a high-speed camera, a dryer, and a water purification system. The equipment used in the experiment is shown in Table 1.

The reagents used in the experiment are shown in Table 2.

### 2.2. Experimental

**Figure 1:**
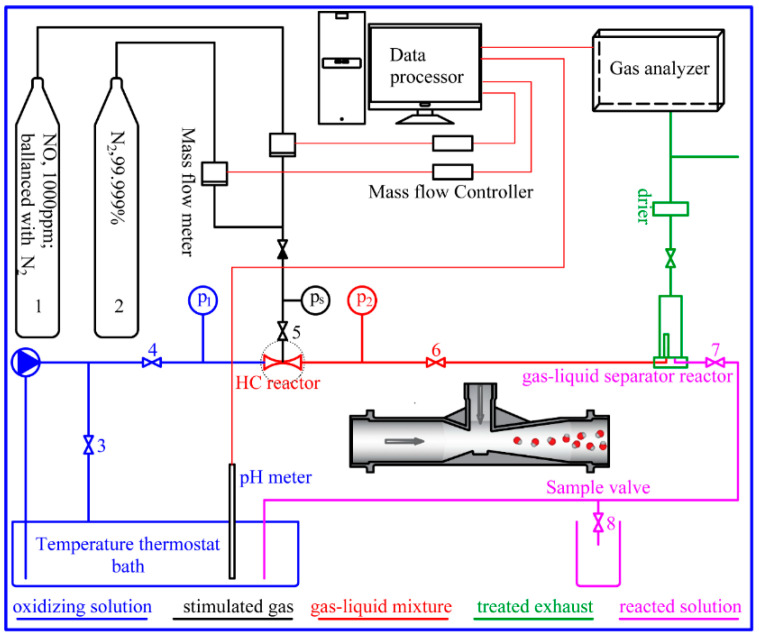
Schematic diagram of the experimental setup.

As shown in Figure 1, the experimental setup was made of simulated gas, the HC reactor, the NaClO_2_ solution, and the flue gas analyzer. The numbers 1 and 2 represent gas cylinders, and the numbers 3–8 represent valves. Different colors are used to depict the solution or gas in different states. The black, blue, red, green, and pink lines represent the simulated gas, NaClO_2_ solution, gas–liquid mixture solution, treated exhaust, and reacted solution, respectively. Mass flow controllers regulated the flow rate of the simulated gas. The temperature of the NaClO_2_ solution (3.0 L) was controlled by the thermostat bath. The differential pressure ∆P was regulated by valves 4 and 6.

In this study, the NaClO_2_ solution was drawn from the thermostat bath through the pump. When the NaClO_2_ solution flowed through the HC reactor at high speed, a low-pressure suction was created at the throat of the HC reactor, drawing the NO mixture. The gas–liquid mixture solution was separated by a gas–liquid separator. After being dried, the treated gas entered the flue gas analyzer for measurement. Simultaneously, the reacted solution flowed back into the thermostat bath through valve 7.

### 2.3. Nomenclature and Calculation of Removal Efficiency

The nomenclature and notation used in this study are shown in Table 3.

NO_x_ concentration in the treated gas is calculated as follows:

(1) $$C_{{\mathrm{NO}}_{x},\;\mathrm{out}}{=C}_{\mathrm{NO},\;\mathrm{out}}{+C}_{{\mathrm{NO}}_{2},\;\mathrm{out}}$$

The removal efficiencies of NO and NO_x_ can be calculated by the following equations:

(2) $$\eta_{\mathrm{NO}}=\frac{C_{\mathrm{in}}{\;-\;C}_{\mathrm{NO},\;\mathrm{out}}}{C_{\mathrm{in}}}$$

(3) $$\eta_{{\mathrm{NO}}_{x}}=\frac{C_{\mathrm{in}}\;{-\;C}_{{\mathrm{NO}}_{x},\;\mathrm{out}}}{C_{\mathrm{in}}}$$

where $C_{\mathrm{in}}$ is the concentration of NO in simulated gas. $C_{\mathrm{NO},\;\mathrm{out}}$ and $C_{{\mathrm{NO}}_{x},\;\mathrm{out}}$ are the concentrations of NO and NO_x_, respectively.

### 2.4. Measurement of Gas Concentration and pH

First, high-purity N_2_ is used to clean the oxygen (O_2_) in the experiment. The experiment started when the O_2_ content dropped to 0.00%. Next, the pH meter and flue gas analyzer record the data regularly, with the counting interval uniformly set to 5 s. When valve 5 is opened, the simulated NO gas is introduced into the system, and data recording is initiated.

## 3. Results and Discussion

### 3.1. NO Removal Enhanced by HC Mechanism

#### 3.1.1. Effect of HC on NO Removal

**Figure 2:**
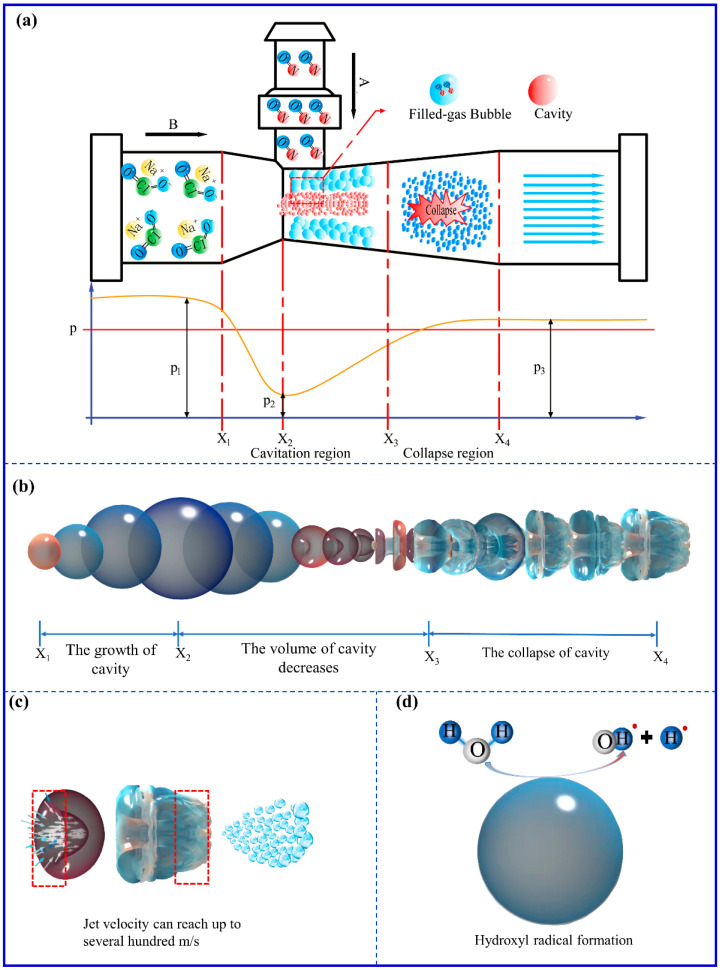
Schematic diagram of HC effect in the HC reactor. (**a**) The operation diagram of the HC reactor. (**b**) The process of cavitation of bubbles from generation to collapse. (**c**) Mechanical effect and (**d**) chemical effect.

Cavitation is the generation, growth, and collapse of cavities when the local pressure in the liquid is lesser than the saturated vapor pressure at the local temperature. As illustrated in Figure 2a, the NaClO_2_ solution moving at high speed enters the HC reactor from A and forms a low suction pressure. The NaClO_2_ solution with dissolved NO mixture forms local cavities at low pressure. According to Gogate’s research, the pressure at the moment of cavitation is generally lower than the saturated vapor pressure at the corresponding temperature [40]. Under low pressure, the cavitation liquid film tends to evaporate inward, thereby balancing the pressure difference between the interior and exterior of the cavities. As the pressure decreases further, the bubble expands rapidly. The cavity is continuously filled with molecules of gas evaporated from the liquid film. At X_2_–X_3_ in Figure 2a, when the NaClO_2_ solution flows through the throat of the HC reactor, the flow area becomes more significant, and the pressure on the NaClO_2_ solution can recover rapidly. As shown in Figure 2b, the volume of cavities decreases continuously under the restoring pressure. Since the compression process of the cavity is extremely short, it can be considered an adiabatic compression process. Rapid compression raises the temperature of the cavities sharply. It forms hot spots with high local temperature and pressure of 5000–15,000 K [41,42,43,44,45] and 100–500 MPa [46,47,48], respectively, ultimately leading the cavity to collapse. As shown in Figure 2c, the collapse of the cavity results in the formation of many tiny bubbles and microjets. The cavitation process promotes chemical reactions through mechanical, thermal, and chemical effects, strengthening NO_x_ removal.

As shown in Figure 2d, under the cavitation conditions, ^•^OH and ^•^H radicals are produced by the pyrolysis of water molecules [49] (as given by Equation (4)).

(4) H2O → OH•+H•

The ^•^OH radicals have a strong oxidation capacity with a redox potential of 2.80 eV [50]. Additionally, NO or NO_2_ may react with ^•^OH either inside or on the surface of the cavities, finally oxidizing to nitric acids (HNO_3_) and nitrous (HNO_2_) [34] (as given by Equations (5)–(7)).

(5) NO+OH• → HNO2

(6) NO+OH• → NO2+H•

(7) NO2+OH• → HNO3

Additionally, ^•^H radicals have an extremely strong reduction capacity and may react with NO or NO_2_ (as given by Equations (8) and (9)).

(8) H•+NO → N•+OH•

(9) NO2(aq)+H• → NO+OH•

In a previous study, the size of bubbles produced at the outlet of the HC and bubbling reactors were compared. It was found that the size of the bubbles in the HC reactor (0.62 mm) was far smaller than those in the bubbling reactor (23.19 mm) [34]. As shown in Figure 2a, the low suction pressure is generated at the throat of the HC reactor. the HC reactor creates low suction pressure in the throat, drawing NO from B. Consequently, the flowing NO was cut by the NaClO_2_ solution flowing at high speed and forming many gas-filled bubbles. The gas-filled bubbles are formed at low pressure, and when the HC reactor’s restoring pressure compresses them, their volumes become smaller (0.50–1.50 mm). Furthermore, since the gas-filled bubbles are slowly compressed, they cannot collapse. However, the small space inside them increases the collision between NO and ^•^OH or ClO_2_, which is conducive to the gas-phase chemical reaction. Additionally, compression of the gas-filled bubbles increases their temperature. Consequently, it increases the impact speed and frequency of NO molecules on the surface of the gas-filled bubbles, enhancing the gas–liquid mass transfer [51,52].

#### 3.1.2. Effect of ∆P on NO Removal

Furthermore, the differential pressure ∆P was adjusted to promote the occurrence of cavitation. The cavitation number C_v_ decreases when ∆P increases. As C_v_ decreases, the cavitation intensity increases. Meanwhile, more reactive radicals may be generated by a higher cavitation intensity which is conducive to NO_x_ removal. Additionally, C_v_ is defined as follows:

(10) $$C_{v}=\frac{2\left(P_{3}-P\right)}{{\rho V}^{2}}$$

where, $P_{3}$, P, V, and ρ denote the outlet pressure of the HC reactor, the vapor pressure of the liquid at saturation temperatures, the velocity of the liquid at the HC reactor throat, and the liquid density, respectively. Furthermore, ideally, cavitation occurs at C_v_ ≤ 1. However, since the introduction of the NO mixture in this study causes the solution to contain dissolved gas, cavitation occurs at C_v_ > 1 [17,53].

**Figure 3:**
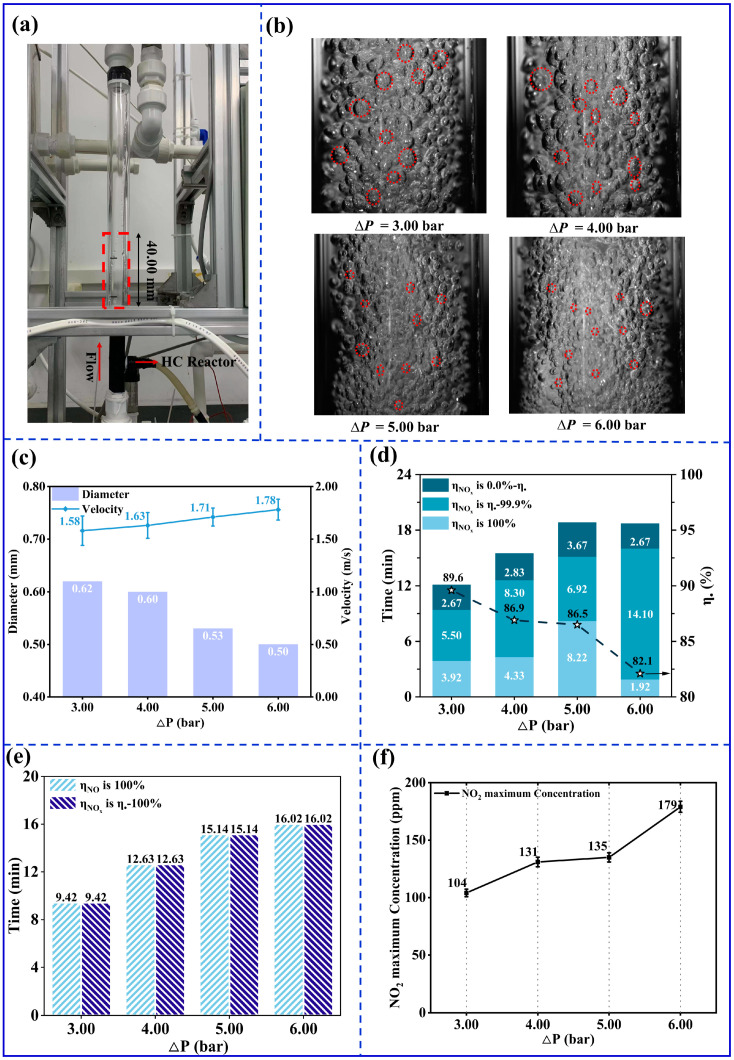
The influence of ∆P of HC reactor. (NO concentration: 1000 ppmv; NaClO_2_ concentration: 1.00 mmol/L; initial pH: 3.50; gas flow: 1.0 L/min; reaction temperature: 45.0 °C; total solution volume: 3.0 L; and ∆P: 3.00 bar, 4.00 bar, 5.00 bar, and 6.00 bar). (**a**) Schematic diagram of the HC reactor connected to a transparent acrylic tube. (**b**) At different ∆P, photographs of the gas-filled bubbles at the outlet were captured by FASTCAM Mini UX50 high-speed camera (frame rate: 8000 fps, and shutter speed: 1/20,000 s). (**c**) Bubble diameter and velocity versus ∆P. (**d**) The duration of NO_x_ removal efficiency = 0−100% versus ∆P. (**e**) The duration of *η*_NOx_ = *η*_●_ − 100% and *η*_NO_ = 100% versus ∆P. (**f**) The outlet maximum NO_2_ concentration versus ∆P.

A transparent acrylic tube (Length: 300.00 mm, Outside diameter: 30.00 mm) was connected to the HC reactor to observe the gas-filled bubbles at the outlet, as shown in Figure 3a. Then a high-speed camera was used to capture the gas-filled bubbles in the 40.00 mm area of the acrylic tube. As illustrated in Figure 3b, as ∆P increases, the size of the gas-filled bubbles at the outlet decreases, and they are gathered more densely. As shown in Figure 3c, the diameters of the gas-filled bubbles were about 0.62 mm with ∆P = 3.00 bar, while they were 0.53 mm with ∆P = 5.00 bar. The surface area and the volume of gas-filled bubbles with ∆P = 5.00 bar were 4.60 and 0.36 times the amount of their equivalents with 3.00 bar. A higher ∆P promotes mixing gas and liquid to increase the contact area. Therefore, the chemical reaction rate accelerates with increasing ∆P for a certain time. A higher ∆P also increases liquid velocity, reducing the overall chemical reaction time. As shown in Figure 3c, when ∆P increased from 3.00 bar to 6.00 bar, the velocity of the gas-filled bubbles increased by 0.20 m/s, and the contact time between gas and liquid reduced by 0.20 s.

Furthermore, the increase in ∆P leads to an increased rate of chemical reaction and shortened reaction time, and this competitive effect affects the duration of the NO_x_ removal efficiency, *η*_NOx_. As illustrated in Figure 3d, as ∆P increases, the time of *η*_NOx_ = 100%, *Tη*_NOx,100%_, first increases and then decreases. When ∆P was 3.00 bar, *Tη*_NOx,100%_ was 3.92 min. *Tη*_NOx,100%_ was maximum (8.22 min) and minimum (1.92 min) when ∆P was 5.00 bar and 6.00 bar, respectively. Therefore, the competitive effect was balanced when ∆P was 5.00 bar.

Furthermore, when *η*_NOx_ is in the range of *η*_●_−99.9%, only NO_2_ is detected in the treated exhaust. Additionally, the oxidation capacity of the NaClO_2_ solution still keeps 100% NO removal efficiency. The highest NO_2_ concentration in the treated gas is reached when *η*_NOx_ is *η*_●_. When ∆P was 3.00 bar, 4.00 bar, 5.00 bar, and 6.00 bar, *η*_●_ was equal to 89.6%, 86.9%, 86.5%, and 82.1%, and the maximum NO_2_ concentration was equal to 104 ppmv, 131 ppmv, 135 ppmv, and 179 ppmv, as shown in Figure 3d,f, respectively. The maximum NO_2_ concentration at ∆P = 5.00 bar was 135 ppmv, which is higher than that at ∆P = 3.00 bar. When the NO_2_ concentration reaches the maximum, *η*_NO_ decreases from 100%. At that moment, the NaClO_2_ solution cannot oxidize NO completely. At ∆P = 5.00 bar, *Tη*_NO,100%_ (15.14 min) was 5.58 min longer than *Tη*_NO,100%_ (9.42 min) at ∆P = 3.00 bar, as shown in Figure 3c. When the NO_2_ concentration reaches the maximum, the NaClO_2_ consumption at ∆P = 5.00 bar was larger than that at ∆P = 3.00 bar. Therefore, the maximum concentration of NO_2_ increased. When ∆P = 6.00 bar, *Tη*_NOx,100%_ was only 1.92 min, and the maximum concentration of NO_2_ (179 ppmv) was reached at the 16th min. Therefore, there was a significant increase in the maximum NO_2_ concentration.

As illustrated in Figure 3e, *Tη*_NO,100%_ was the longest for ∆P *=* 6.00 bar. The primary reason for the increase in the NO_2_ concentration was that large amounts of ClO_2_ escape due to a high ∆P. The high ∆P results in lower suction and pressure of the gas-filled bubbles, which is more conducive for vaporizing the liquid into the bubbles. NaClO_2_ generates adequate ClO_2_ rapidly for initial pH of 3.50 (as given in Equation (11)). Therefore, at the high ∆P, ClO_2_ in the liquid phase is more likely to be vaporized into and discharged together with the gas-filled bubbles.

(11) $${5\mathrm{ClO}}_{2}^{-}{+H}^{+}\;\to {\;4\mathrm{ClO}}_{2}{+\mathrm{Cl}}^{-}{+2H}_{2}O$$

Additionally, a high ∆P shortens the reaction time. Furthermore, the absorption of NO_2_ becomes insufficient due to the short contact time between gas and liquid. NO_2_ requires time to be converted to N_2_O_3_ and N_2_O_4_ (as given in Equations (12) and (13)), which were dissolved by the liquid phase (as given in Equations (14) and (15)) [20,54,55]. It is generally accepted that the increase in nitrogen valency increases the solubility of gaseous nitrogen in the aqueous phase [56]. Therefore, the short reaction time would inhibit this process, and the absorption of NO_2_ would become insufficient.

(12) $${\mathrm{NO}+\mathrm{NO}}_{2}\;\to {\;N}_{2}O_{3}$$

(13) $${2\mathrm{NO}}_{2}\;\to {\;N}_{2}O_{4}$$

(14) $$N_{2}O_{3}{+H}_{2}O\;\to {\;2\mathrm{HNO}}_{2}$$

(15) $$N_{2}O_{4}{+H}_{2}O\;\to {\;\mathrm{HNO}}_{2}{+\mathrm{HNO}}_{3}$$

Summing up, for ∆P = 5.00 bar, the influence of increased reaction rate and shortened reaction time reached a good balance. Furthermore, the maximum NO_2_ concentration was only 135 ppmv, and *Tη*_NOx,100%_ was the maximum (8.22 min). Therefore, ∆P = 5.00 bar was used as the experimental optimal ∆P.

### 3.2. Effect of Initial pH of NaClO_2_ Solution on NO Removal

**Figure 4:**
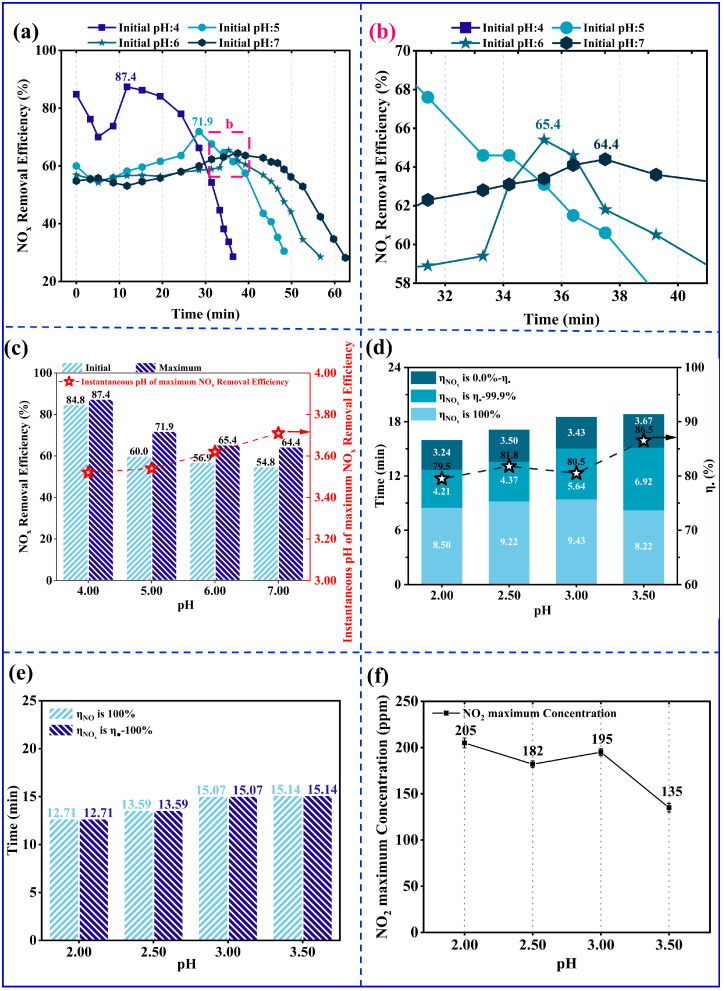
The impact of pH. (NO concentration: 1000 ppmv; NaClO_2_ concentration: 1.00 mmol/L; gas flow: 1.0 L/min; ∆P: 5.00 bar; reaction temperature: 45.0 °C; total liquid volume: 3.0 L; initial pH: 2.00, 2.50, 3.00, 3.50, 4.00, 5.00, 6.00, and 7.00.) (**a**) At initial pH = 4.00−7.00, the efficient removal NO_x_ versus time. (**b**) The enlarged drawing of (**a**). (**c**) At initial pH = 4.00−7.00, the initial and maximum NO_x_ removal efficiency versus the initial pH. (**d**) At initial pH = 2.00−3.50, the duration of NO_x_ removal efficiency = 0−100% versus the initial pH. (**e**) At initial pH = 2.00−3.50, the duration of *η*_NOx_ = *η*_●_ − 100% and *η*_NO_ = 100% versus the initial pH. (**f**) The outlet maximum NO_2_ concentration versus the initial pH.

According to the Nernst equation, the reduction potential of NaClO_2_ decreases as the pH increases. However, since pH affects the generation of NaClO_2_ to ClO_2_, there is an optimal pH for NO_x_ removal [13,57,58]. Yang et al. [59] and Adewuyi et al. [58] suggested removing NO_x_ by NaClO_2_ in neutral or slightly acidic conditions. Therefore, the experiments were first performed at an initial pH of 4.00−7.00 in this study. As the initial pH decreases, the *η*_NOx initial_ increases. As illustrated in Figure 4a,b, for the initial pH range of the solution of 4.00−7.00, the initial NO_x_ removal efficiency *η*_NOx initial_ is 84.8−54.8%. Since, at this time, the amount of ClO_2_ generated by NaClO_2_ was not enough to oxidize NO_x_ completely, the *η*_NOx initial_ could not reach 100%. Furthermore, as the solution absorbs more NO_x_, its pH gradually decreases, and *η*_NOx_ reaches its maximum value *η*_NOx max_. When the initial pH was 4.00, 5.00, 6.00, and 7.00, the values of *η*_NOx max_ were 87.4%, 71.9%, 65.4%, and 64.4%. The increase in *η*_NOx max_ is by 2.6%, 11.9%, 8.5%, and 9.6%, respectively, compared to *η*_NOx initial_. As shown in Figure 4c, the instantaneous pH range of achieving *η*_NOx max_ is 3.50−3.70, according to the experimental results. The authors of this study were of the opinion that adjusting the initial pH to 3.50−3.70 may improve *η*_NOx initial_, so the experiment was carried out for initial pH = 3.50. Subsequently, it was shown that *η*_NOx initial_ could reach 100% for the initial pH = 3.50. When the initial pH of the NaClO_2_ solution is 4.00 − 7.00, the NO_x_ of treated emissions consists of NO and NO_2_. NO was not completely oxidized, and NO_2_ was not completely absorbed, resulting in the *η*_NOx initial_ being less than 100%. The reduction of initial pH could significantly improve the oxidation capacity of the NaClO_2_ solution. Gong et al. [60] explained that the NO removal efficiency increased with the decrease of pH. A 100% removal efficiency of NO could be achieved when the pH was below 2.5. In addition, when the initial pH of the NaClO_2_ solution is 4.00−7.00, due to the reduced amount of ClO_2_ generated, NO_2_ was not completely absorbed. Song et al. [37] carried out research on the removal of NO_2_ by H_2_O_2_, NaS_2_O_8_, NaClO_2_, and ClO_2_ under HC conditions, and reported that ClO_2_ has a higher oxidation selectivity for NO_2_ compared with NaClO_2_. When the initial pH was 4.00−7.00, the ClO_2_ generated per unit of time was small [61]. When the experiment was carried out for initial pH = 3.50, the amount of ClO_2_ generated per unit of time was more significant [62]. Therefore, *η*_NOx initial_ could reach 100% for the initial pH = 3.50.

Furthermore, the reduction of initial pH could significantly improve the NO_x_ removal efficiency. Experiments with an initial pH of 2.00−3.50 were carried out in this study to explore further the influence of initial pH on removing NO based on HC combined with NaClO_2_, so an acidic oxidation mechanism was followed between NO and NaClO_2_ [63]. Therefore, NO is removed by reacting with ClO_2_^−^ (as given by Equation (16)) or ClO_2_ (as given by Equation (17)). In addition, ^•^OH (as given by Equations (5) and (6)) and ^•^H (as given by Equation (8)) will also promote the removal of NO. As shown in Figure 4d, when the initial pH was 2.00, 2.50, 3.00, and 3.50, *η*_NOx initial_ reached 100% and maintained this value for more than 8 min. Furthermore, this depends on the rapid decomposition of NaClO_2_ to generate ClO_2_ in acidic conditions (as given in Equation (11)). A significantly low value of pH shortens *Tη*_NOx,100%_. When the initial pH was 2.00, 2.50, and 3.50, *Tη*_NOx,100%_ was 8.50 min, 9.22 min, and 9.43 min, respectively, as shown in Figure 4d. The reason for this phenomenon may be the escape of excess ClO_2_ from the liquid phase [64]. The reaction rate of Equation (11) may be influenced by the ClO_2_^−^ and H^+^ concentrations. The reaction rate of Equation (11) is faster for higher ClO_2_^−^ and H^+^ concentrations, and more ClO_2_ is produced per unit of time. *Tη*_NOx,100%_ was the maximum for initial pH = 3.00, and the production of ClO_2_ was sufficient for NO removal (1000 ppmv, 1.0 L/min) in a unit of time. However, ClO_2_ was overproduced for initial pH of 2.00 and 2.50. The excessive ClO_2_ vaporized into gas-filled bubbles and discharged together with them. Thus, NaClO_2_ consumption was accelerated, leading to a reduction of *Tη*_NOx,100%_.

(16) $${2\mathrm{NO}+\mathrm{ClO}}_{2}^{-}\;\to {\;2\mathrm{NO}}_{2}{+\mathrm{Cl}}^{-}$$

(17) $${2\mathrm{ClO}}_{2}{+5\mathrm{NO}+H}_{2}O\;\to {\;5\mathrm{NO}}_{2}{+2H}^{+}{+2\mathrm{Cl}}^{-}$$

When *η*_NOx_ was in the interval of *η*_●_−99.9%, the NaClO_2_ solution concentration decreased, and the reaction rate of Equation (11) became slow. In this case, the influence of ClO_2_ escaped on the duration of the interval of *η*_NOx_ became smaller, and the remaining NaClO_2_ in the solution had a more significant influence on the duration of the interval. The amount of NaClO_2_ remaining in the solution became lesser as the duration of the interval lengthened. When the initial pH was 2.00, 2.50, 3.00, and 3.50, the duration of the interval of *η*_NOx_ was 4.21 min, 4.37 min, 5.64 min, and 6.92 min, respectively, as shown in Figure 4d. Simultaneously, the remaining NaClO_2_ in the solution also affected the maximum NO_2_ concentration in the treated gas. As shown in Figure 4f, the maximum NO_2_ concentration generally declines. When the initial pH was 2.00, 2.50, 3.00, and 3.50, the maximum NO_2_ concentration was 205 ppmv, 182 ppmv, 195 ppmv, and 135 ppmv, respectively. Chin et al. [65] and Brogren et al. [66] explained that 60−80% of the NO_2_ generated in the reaction can be removed by Equations (18)–(22).

Additionally, ^•^OH (Equation (7)) and ^•^ClO (Equations (23)–(25)) also promoted NO_2_ absorption [37]. Therefore, this may be the reason for the complete NO_2_ removal when *η*_NOx_ was 100%. When *η*_NOx_ was in the interval of *η*_●_−99.9%, the NaClO_2_ solution concentration decreased, which inhibited NO_2_ removal by Equations ((22)–(25)). In this case, the generated NO_2_ may be removed by the hydrolysis of NO_2_ (as given by Equations (18) and (19)), oxidative absorption of ClO_2_^−^ (as given by Equations (20) and (21)), and ^•^OH (as given by Equation (7)). Therefore, this could also explain that, as NaClO_2_ in solution decreased, the maximum NO_2_ concentration was only 205 ppmv.

(18) $${3\mathrm{NO}}_{2}{+H}_{2}O\;↔{\;2\mathrm{HNO}}_{3}+\mathrm{NO}$$

(19) $${2\mathrm{NO}}_{2}{+H}_{2}O\;↔{\;\mathrm{HNO}}_{3}{+\mathrm{HNO}}_{2}$$

(20) $${2\mathrm{NO}}_{2}{+\mathrm{ClO}}_{2}^{-}\;\to {\;2\mathrm{NO}}_{3}^{-}{+\mathrm{Cl}}^{-}$$

(21) $${\mathrm{NO}}_{2}{+\mathrm{ClO}}_{2}^{-}\;\to {\;2\mathrm{NO}}_{3}^{-}{+\mathrm{ClO}}^{-}$$

(22) $${2\mathrm{NO}}_{2}{+\mathrm{ClO}}_{2}\;\to {\;2\mathrm{NO}}_{3}^{-}{+\mathrm{Cl}}^{-}$$

(23) ClO2(g) →cavitation ClO•(g)+O•(g)

(24) ClO•+NO2 → ClONO2

(25) $${\mathrm{ClONO}}_{2}{+H}_{2}O\;\to {\;\mathrm{HOCl}+\mathrm{HNO}}_{3}$$

When the initial pH was 3.50, 100% NO_x_ removal efficiency was maintained for 8.22 min. Subsequently, NO_2_ was detected in the treated gas, but the oxidation capacity of the NaClO_2_ solution could still maintain 100% NO removal efficiency for 6.92 min. *η*_NOx_ decreased from *η*_●_ to 0.0% (*η*_●_ = 86.5%), the NO concentration increased from 0 ppmv to 1000 ppmv, and NO_2_ concentration rapidly decreased from 135 ppmv to 0 ppmv in the next 3.67 min. Therefore, this indicated that the decreased NaClO_2_ solution concentration led to the loss of oxidation capacity for NO removal. Therefore, for the NO_x_ removal by NaClO_2_ solution under acidic conditions, the fundament was the rapid activation of ClO_2_, and the increasing *Tη*_NOx,100%_ required improved NO_2_ absorption. A lower value of initial pH increased *Tη*_NOx,100%_, but a large amount of escaping ClO_2_ led to a reduced oxidation capacity and a higher NO_2_ concentration in the solution. The lower pH can also be a severe concern for the corrosion of the experimental equipment. Therefore, the optimal initial pH of the solution was taken as 3.50 in this study.

### 3.3. Effect of Reaction Temperature on NO Removal

**Figure 5:**
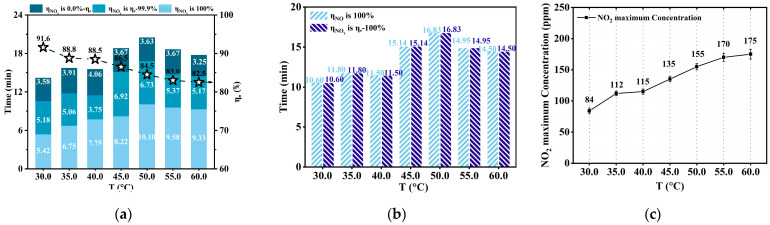
The impact of reaction temperature. (NO concentration: 1000 ppmv; NaClO_2_ concentration: 1.00 mmol/L; initial pH: 3.50; gas flow: 1.0 L/min; ∆P: 5.00 bar; total liquid volume: 3.0 L; reaction temperature: 30.0 °C, 35.0 °C, 40.0 °C, 45.0 °C, 50.0 °C, 55.0 °C, and 60.0 °C); (**a**) The duration of NO_x_ removal efficiency = 0−100% versus reaction temperature. (**b**) The duration of *η*_NOx_ = *η*_●_ − 100% and *η*_NO_ = 100% versus reaction temperature. (**c**) The outlet maximum NO_2_ concentration versus reaction temperature.

The reaction temperature significantly influences the dissolution and diffusion of molecules or ions in the NaClO_2_ solution. Additionally, the change in the reaction temperature would affect the change in the saturated vapor pressure of the solution, affecting the cavitation. According to the Arrhenius law, a high temperature promotes ion diffusion and accelerates chemical reactions [10]. The high temperature promotes the thermal decomposition of NaClO_2_ to generate ClO_2_ (as given in Equation (11)) [67]. As the reaction temperature increased, *Tη*_NOx,100%_ first increased and then decreased. When the reaction temperature was 30.0 °C, 35.0 °C, 40.0 °C, 45.0 °C, 50.0 °C, 55.0 °C, and 60.0 °C, *Tη*_NOx,100%_ was 5.42 min, 6.75 min, 7.75 min, 8.22 min, 10.10 min, 9.58 min, and 9.33 min, respectively, as shown in Figure 5a. Therefore, the increase in temperature had the same influence on *Tη*_NO,100%_. As shown in Figure 5b, *Tη*_NO,100%_ was the shortest (10.60 min) for 30.0 °C reaction temperature. Additionally, *Tη*_NO,100%_ was the longest (16.83 min) for 50.0 °C reaction temperature. However, *Tη*_NO,100%_ decreased to 14.50 min for 60.0 °C reaction temperature. The decrease in the *Tη*_NO,100%_ value indicated that NO could not be fully oxidized. This was because the high temperature accelerated the thermal decomposition of NaClO_2_ into ClO_2_, which led to the consumption of NaClO_2_ in the solution.

However, the high temperature decreases the solubility of NO_x_ or ClO_2_. Additionally, it enhances the mass-transfer resistance between gas and liquid, resulting in a decreased mass transfer of NO from the gas to the liquid phase. Therefore, when the reaction temperature exceeded 50.0 °C, NO_x_ absorption was inhibited due to the decrease in NO_x_ solubility. Therefore, as temperature increased, *Tη*_NO,100%_ first increased and then decreased. An increase in temperature would also increase the maximum NO_2_ concentration in the treated gas. Furthermore, when the temperature increased from 30.0 °C to 60.0 °C, the maximum NO_2_ concentration increased from 84 ppmv to 175 ppmv, as shown in Figure 5c. Nitrites of the solution decomposed into NO_2_ at higher reaction temperatures (as given in Equation (26)) [67], which may be one of the reasons for an increase in the maximum NO_2_ concentration with the increase in temperature.

(26) $${2\mathrm{HNO}}_{2}\;\to {\;\mathrm{NO}+\mathrm{NO}}_{2}{+H}_{2}O$$

In addition to this, a temperature change will cause a change in cavitation intensity. The influence of temperature on cavitation intensity is mainly through viscous and thermodynamic effects [32]. As temperature increases and viscosity decreases, the Reynolds number increases proportionally. The generation of turbulence effects increases the intensity of cavitation. Temperature increases the vapor pressure, making it easier for the NaClO_2_ solution to evaporate and accelerating the formation of cavities [68]. Therefore, the values of *Tη*_NOx,100%_ and *Tη*_NO,100%_ keep increasing as the temperature rises from 30 °C to 50 °C. However, too high of a temperature will have a delay effect on cavitation. Brennen quantifies the delays of cavitation with the thermodynamic parameter Σ [69], as follows:

(27) $$\Sigma =\frac{{\left(\rho_{V}\;L\right)}^{2}}{\rho_{l}^{2}c_{p,l}T_{\infty }\sqrt{\alpha_{l}}}$$

where $T_{\infty }$ is the test temperature, $\rho_{V}$ is the vapor density, $\rho_{l}$ is the liquid density, L is the evaporative latent heat, $c_{p,l}$ is the constant pressure specific heat of the liquid, and $\alpha_{l}$ is the thermal diffusivity of the liquid.

The ∑ parameter depends only upon the liquid’s temperature; thus, various liquids can be compared to each other regarding the thermal delay. Hattori et al. [70] reported that the thermodynamic effect becomes significant when the thermodynamic parameter ∑ = 100 m/s^3/2^. For water, the applicable range is 50 °C and 55 °C. When the temperature in this study exceeds 50 °C, the thermodynamic effect significantly retards the development of cavitation. At this point, the increase in vapor pressure tends to evaporate the liquid, causing cavities to merge and reducing the number of individual cavitation structures [68]. The delay in cavitation causes a reduction in cavitation intensity. Therefore, the chemical effect of cavitation will also be weakened, and the production of ^•^OH and ^•^H (as given in Equation (4)) and ^•^ClO (as given in Equation (23)) will be reduced, which is not conducive to the removal of NO_x_ (as given in Equations (5)–(9) and (24) and (25)).

### 3.4. Effect of NaClO_2_ Concentration on NO Removal

**Figure 6:**
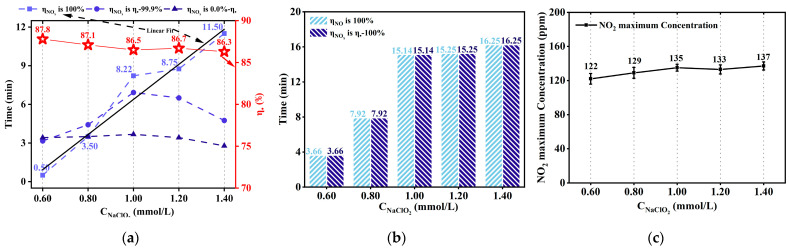
The impact of NaClO_2_ concentration. (NO concentration: 1000 ppm; total liquid volume: 3.0 L; ∆P: 5.00 bar; initial pH: 3.50; gas flow: 1.0 L/min; reaction temperature: 45.0 °C; NaClO_2_ concentration: 0.60 mmol/L, 0.80 mmol/L, 1.00 mmol/L, 1.20 mmol/L, 1.40 mmol/L). (**a**) The duration of NO_x_ removal efficiency = 0 − 100% versus NaClO_2_ concentration. (**b**) The duration of *η*_NOx_ = *η*_●_ − 100% and *η*_NO_ = 100% versus NaClO_2_ concentration. (**c**) The outlet maximum NO_2_ concentration versus NaClO_2_ concentration.

The increased concentration of NaClO_2_ enhanced the mass transfer effect between the gas and liquid phases. As illustrated in Figure 6a, when the NaClO_2_ concentration was 0.60 mmol/L, *η*_NOx_ reached 100%. However, the duration was only 0.50 min. A short duration is not conducive to observing the complete reactive trend of NO_x_ removal by HC. Furthermore, when the concentration of NaClO_2_ increased from 0.60 mmol/L to 1.40 mmol/L, *Tη*_NOx,100%_ increased from 0.50 min to 11.50 min. Therefore, *Tη*_NOx,100%_ is linear with the NaClO_2_ concentration (as given in Equation (28)), and the corresponding slope and the intercept of the straight line are 13.625 ± 1.32 and 7.24 ± 1.37, respectively.

y = (13.625 ± 1.32) x − (7.24 ± 1.37)(28)

Furthermore, when the NaClO_2_ concentration was 1.00 mmol/L, *Tη*_NOx,100%_ was 8.22 min, which was 1.84 min higher than the predicted value of 6.38 min in Equation (28). As the concentration of NaClO_2_ increases, the amount of ClO_2_ produced will also increase. However, when the NaClO_2_ concentrations were 0.60 mmol/L, 0.80 mmol/L, 1.20 mmol/L, and 1.40 mmol/L, the corresponding *Tη*_NOx,100%_ values were lower than the predicted values, as shown in Figure 6.

In addition to this, as illustrated in Figure 6b, when the concentrations of NaClO_2_ were 1.20 mmol/L, and 1.40 mmol/L, *Tη*_NO,100%_ values were 15.25 min and 16.25 min, respectively. Furthermore, compared with the *Tη*_NO,100%_ value of 15.14 min for NaClO_2_ concentration of 1.00 mmol/L, they showed an increase of 0.11 min and 1.11 min, respectively. However, when the concentration exceeded 1.00 mmol/L, *Tη*_NO,100%_ values did not increase significantly. Additionally, the increase in NaClO_2_ concentration did not significantly reduce the average concentration of NO_2_ (135 ± 13 ppmv), as shown in Figure 6c. The maximum concentration of NO_2_ was 137 ppmv when the NaClO_2_ concentration was 1.20 mmol/L, which was 2 ppmv higher than when the NaClO_2_ concentration was 1.00 mmol/L. NO_2_ was transported by the escape of ClO_2_, resulting in a higher concentration of NO_2_ [35]. Therefore, the optimal NaClO_2_ concentration was considered as 1.00 mmol/L in this study.

## 4. Conclusions

Detailed experiments were carried out to study the influence of various parameters on NO removal efficiency, including the ∆P of the HC reactor, the initial pH, the reaction temperature, and the concentration of NaClO_2_. The experimental results showed that removing NO from ship exhaust based on HC using NaClO_2_ solution was a valid method. The advantages of this novel method were low NaClO_2_ concentration and high NO_x_ removal efficiency. The NO_x_ removal efficiency reached 100% for the NaClO_2_ concentration of 0.60 mmol/L. The HC reactor could generate many gas-filled bubbles with small volumes, which was conducive to enhancing the contact area between liquid and gas to accelerate the reaction rate. The reduction of initial pH could significantly improve the oxidation capacity of the NaClO_2_ solution. *η*_NOx initial_ was below 100% for the initial pH = 4.00–7.00. When the initial pH ≤ 3.50, *η*_NOx initial_ reached 100% and was maintained for more than 8 min. The fundamentals for NO_x_ removal by NaClO_2_ solution under acidic conditions was the rapid activation of ClO_2_, and the increasing *Tη*_NOx,100%_ required improved NO_2_ absorption. Additionally, ^•^OH and ^•^ClO produced by HC promoted the NO_2_ absorption, which may be one of the reasons for complete NO_2_ removal when *η*_NOx_ was 100%.

## Figures and Tables

**Table 1:** The experiment instruments.

Equipment	Equipment Type	Manufacturer
Flue gas analyzer	Gasboard-3000UV	Cubic-Ruiyi Co., Ltd., Wuhan, China
High-speed camera	FASTCAM Mini UX50	Photron, San Diego, CA, USA
pH meter	S210	Mettler-Toledo Instruments Co., Ltd., Columbus, OH, USA
Dryer	XX100A-03	Suzhou Xiaoxiong Electric Co., Ltd., Suzhou, China
HC reactor	Model 287	Mazzei Injector Company, LLC, Bakersfield, CA, USA
Milli-Q Plus water purification system	Master-Q15	Millipore, Burlington, MA, USA

**Table 2:** Experimental reagents.

Reagent	Purity (Concentration)	Manufacturer
NaClO_2_	AR	Sinopharm Chemical Reagent Co., Shanghai, China
HCl	36–38%	Sinopharm Chemical Reagent Co.
N_2_	99.999%	Dalian Special Gases Co., Ltd., Baotou, China
NO/N_2_	1000 ppmv	Dalian Special Gases Co., Ltd.

**Table 3:** Nomenclature and Notation.

HC	Hydrodynamic cavitation	*Tη*_NOx,100%_	The time of *η*_NOx_ = 100% (min)
∆P	Differential pressure (bar)	*η*_NOx_	The removal efficiency of NO_x_ (%)
*η*_NO_	The removal efficiency of NO (%)	*η*_●_	The removal efficiency of NO_x_ when NO is initially detected by the flue gas analyzer (%)
*η*_NOx initial_	The initial removal efficiency of NO_x_ with an initial pH of 4 − 7 (%)	*η*_NOx max_	The maximum removal efficiency of NO_x_ with an initial pH of 4 − 7 (%)
*Tη*_NO,100%_	The time of *η*_NO_ = 100% (min)	C_v_	Cavitation number

## Data Availability

Not applicable.

## Supplementary Materials

The following supporting information can be downloaded at: https://www.mdpi.com/article/10.3390/ijerph20043684/s1, Figure S1: The structure and dimensions of HC reactor; Table S1: Factors affecting the NO_x_ removal efficiency by composite NaClO_2_-containing oxidants. Refs. [20,22,29,30,38,39,54,71,72,73,74,75] are cited in Supplementary Materials file.

## References

[1] Maritime Knowledge Centre International Shipping Facts and Figures–Information Resources on Trade, Safety, Security, and the Environment. https://vdocument.in/international-shipping-facts-and-figures-56264a5f6198f.html?page=1.

[2] Zhao J., Wei Q., Wang S., Ren X. (2021). Progress of ship exhaust gas control technology. Sci. Total Environ..

[3] Tong D., Zhang Q., Davis S.J., Liu F., Zheng B., Geng G.N., Xue T., Li M., Hong C.P., Lu Z.F. (2018). Targeted emission reductions from global super-polluting power plant units. Nat. Sustain..

[4] De Marco A., Proietti C., Anav A., Ciancarella L., D’Elia I., Fares S., Fornasier M.F., Fusaro L., Gualtieri M., Manes F. (2019). Impacts of air pollution on human and ecosystem health, and implications for the National Emission Ceilings Directive: Insights from Italy. Environ. Int..

[5] Perera F.P. (2017). Multiple threats to child health from fossil fuel combustion: Impacts of air pollution and climate change. Environ. Health Perspect..

[6] Wang X.C., Klemes J.J., Dong X.B., Fan W.G., Xu Z.H., Wang Y.T., Varbanov P.S. (2019). Air pollution terrain nexus: A review considering energy generation and consumption. Renew. Sustain. Energy Rev..

[7] Hao R., Wang X., Zhao X., Xu M., Zhao Y., Mao X., Yuan B., Zhang Y., Gao K. (2018). A novel integrated method of vapor oxidation with dual absorption for simultaneous removal of SO_2_ and NO: Feasibility and prospect. Chem. Eng. J..

[8] Si M., Shen B., Adwek G., Xiong L., Liu L., Yuan P., Gao H., Liang C., Guo Q. (2021). Review on the NO removal from flue gas by oxidation methods. J. Environ. Sci..

[9] Lehtoranta K., Vesala H., Koponen P., Korhonen S. (2015). Selective Catalytic Reduction Operation with Heavy Fuel Oil: NOx, NH_3_, and Particle Emissions. Environ. Sci. Technol..

[10] (2014). Summary for Policymakers. Climate Change 2013–The Physical Science Basis: Working Group I Contribution to the Fifth Assessment Report of the Intergovernmental Panel on Climate Change.

[11] Zheng M., Reader G.T., Hawley J.G. (2004). Diesel engine exhaust gas recirculation––A review on advanced and novel concepts. Energy Convers. Manag..

[12] Sharif H.M.A., Mahmood N., Wang S., Hussain I., Hou Y.-N., Yang L.-H., Zhao X., Yang B. (2021). Recent advances in hybrid wet scrubbing techniques for NOx and SO_2_ removal: State of the art and future research. Chemosphere.

[13] Wang H., Yuan B., Hao R., Zhao Y., Wang X. (2019). A critical review on the method of simultaneous removal of multi-air-pollutant in flue gas. Chem. Eng. J..

[14] Chen R., Zhang T., Guo Y., Wang J., Wei J., Yu Q. (2021). Recent advances in simultaneous removal of SO_2_ and NOx from exhaust gases: Removal process, mechanism and kinetics. Chem. Eng. J..

[15] Liu Y., Wang Q., Yin Y., Pan J., Zhang J. (2014). Advanced oxidation removal of NO and SO_2_ from flue gas by using ultraviolet/H_2_O_2_/NaOH process. Chem. Eng. Res. Des..

[16] Gogate P.R., Patil P.N. (2015). Combined treatment technology based on synergism between hydrodynamic cavitation and advanced oxidation processes. Ultrason. Sonochem..

[17] Saxena S., Saharan V.K., George S. (2018). Enhanced synergistic degradation efficiency using hybrid hydrodynamic cavitation for treatment of tannery waste effluent. J. Clean. Prod..

[18] Wacławek S., Lutze H.V., Grübel K., Padil V.V.T., Černík M., Dionysiou D.D. (2017). Chemistry of persulfates in water and wastewater treatment: A review. Chem. Eng. J..

[19] Adewuyi Y.G., Khan M.A. (2015). Nitric oxide removal by combined persulfate and ferrous–EDTA reaction systems. Chem. Eng. J..

[20] Liu Z.-H., Xu H.-Z., Li Y.-B., Luo Y., Zhang L.-L., Chu G.-W. (2022). Nox removal from gas mixture intensified by rotating packed bed with NaClO_2_ preoxidation. Chem. Eng. J..

[21] Guo R.-T., Yu Y.-L., Pan W.-G., Ding H.-L., Xin Z.-L., Zhang X.-B., Jin Q., Ding C.-G., Guo S.-Y. (2014). Absorption of NO by Aqueous Solutions of KMnO_4_/H_2_SO_4_. Sep. Sci. Technol..

[22] Hao R., Zhang Y., Wang Z., Li Y., Yuan B., Mao X., Zhao Y. (2017). An advanced wet method for simultaneous removal of SO_2_ and NO from coal-fired flue gas by utilizing a complex absorbent. Chem. Eng. J..

[23] Hao R., Wang X., Liang Y., Lu Y., Cai Y., Mao X., Yuan B., Zhao Y. (2017). Reactivity of NaClO_2_ and HA-Na in air pollutants removal: Active species identification and cooperative effect revelation. Chem. Eng. J..

[24] Hao R., Yang S., Zhao Y., Zhang Y., Yuan B., Mao X. (2017). Follow-up research of ultraviolet catalyzing vaporized H_2_O_2_ for simultaneous removal of SO_2_ and NO: Absorption of NO_2_ and NO by Na-based WFGD byproduct (Na_2_SO_3_). Fuel Process. Technol..

[25] Kang M.S., Shin J., Yu T.U., Hwang J. (2020). Simultaneous removal of gaseous NOx and SO_2_ by gas-phase oxidation with ozone and wet scrubbing with sodium hydroxide. Chem. Eng. J..

[26] Hao R., Mao X., Wang Z., Zhao Y., Wang T., Sun Z., Yuan B., Li Y. (2019). A novel method of ultraviolet/NaClO_2_-NH_4_OH for NO removal: Mechanism and kinetics. J. Hazard. Mater..

[27] Deshwal B.R., Kundu N. (2018). Comparing Acidic Sodium Hypochlorite and Sodium Chlorite Solutions for Controlling Nitrogen Oxides Emission. Environ. Eng. Sci..

[28] Deshwal B.R., Lee S.H., Jung J.H., Shon B.H., Lee H.K. (2008). Study on the removal of NOx from simulated flue gas using acidic NaClO_2_ solution. J. Environ. Sci..

[29] Han Z., Lan T., Han Z.-T., Yang S., Dong J.M., Sun D.D., Yan Z., Pan X., Song L. (2019). Simultaneous Removal of NO and SO_2_ from Exhaust Gas by Cyclic Scrubbing and Online Supplementing pH-Buffered NaClO_2_ Solution. Energy Fuels.

[30] Hao R., Yang S., Yuan B., Zhao Y. (2017). Simultaneous desulfurization and denitrification through an integrative process utilizing NaClO_2_/Na_2_S_2_O_8_. Fuel Process. Technol..

[31] Charpentier J.-C., Drew T.B., Cokelet G.R., Hoopes J.W., Vermeulen T. (1981). Mass-Transfer Rates in Gas-Liquid Absorbers and Reactors. Advances in Chemical Engineering.

[32] Ge M., Sun C., Zhang G., Coutier-Delgosha O., Fan D. (2022). Combined suppression effects on hydrodynamic cavitation performance in Venturi-type reactor for process intensification. Ultrason. Sonochemistry.

[33] Sun X., Liu S., Zhang X., Tao Y., Boczkaj G., Yoon J.Y., Xuan X. (2022). Recent advances in hydrodynamic cavitation-based pretreatments of lignocellulosic biomass for valorization. Bioresour. Technol..

[34] Song L.G., Yang J.G., Yu S.B., Xu M.Y., Liang Y.C., Pan X.X., Yao L. (2019). Ultra-high efficient hydrodynamic cavitation enhanced oxidation of nitric oxide with chlorine dioxide. Chem. Eng. J..

[35] Yang J., Song L., Wei Y., Sui H., Deng C., Zhang B., Lu K., Xu M., Han Z., Pan X. (2022). A novel one-step wet denitration method by hydrodynamic cavitation and chlorine dioxide. J. Environ. Chem. Eng..

[36] Yang J., Song L., Deng C., Sui H., Dionysiou D.D., Han Z., Xu M., Pan X. (2023). A new multi-component marine exhaust cleaning method using combined hydrodynamic cavitation and chlorine dioxide. Sep. Purif. Technol..

[37] Song L., Yang J., Sui H., Wei Y., Deng C., Meng L., Guo F., Han Z., Pan X., Dionysiou D.D. (2023). A novel method based on hydrodynamic cavitation to effectively remove NO_2_. Chem. Eng. J..

[38] Zhao Y., Hao R., Yuan B., Jiang J. (2016). Simultaneous removal of SO_2_, NO and Hg^0^ through an integrative process utilizing a cost-effective complex oxidant. J. Hazard. Mater..

[39] Zhao Y., Guo T.-X., Chen Z.-Y., Du Y.-R. (2010). Simultaneous removal of SO_2_ and NO using M/NaClO_2_ complex absorbent. Chem. Eng. J..

[40] Gogate P.R., Pandit A.B. (2005). A review and assessment of hydrodynamic cavitation as a technology for the future. Ultrason. Sonochemistry.

[41] McNamara W.B., Didenko Y.T., Suslick K.S. (1999). Sonoluminescence temperatures during multi-bubble cavitation. Nature.

[42] Xu H., Glumac N.G., Suslick K.S. (2010). Temperature inhomogeneity during multibubble sonoluminescence. Angew. Chem. (Int. Ed. Engl.).

[43] Flannigan D.J., Suslick K.S. (2005). Plasma formation and temperature measurement during single-bubble cavitation. Nature.

[44] Sun X., You W., Xuan X., Ji L., Xu X., Wang G., Zhao S., Boczkaj G., Yoon J.Y., Chen S. (2021). Effect of the cavitation generation unit structure on the performance of an advanced hydrodynamic cavitation reactor for process intensifications. Chem. Eng. J..

[45] Xuan X., Wang M., You W., Manickam S., Tao Y., Yong Yoon J., Sun X. (2023). Hydrodynamic cavitation-assisted preparation of porous carbon from garlic peels for supercapacitors. Ultrason. Sonochemistry.

[46] Suslick K.S., Eddingsaas N.C., Flannigan D.J., Hopkins S.D., Xu H.X. (2018). The chemical history of a bubble. Accounts Chem. Res..

[47] McNamara W.B., Didenko Y.T., Suslick K.S. (2003). Pressure during sonoluminescence. J. Phys. Chem. B.

[48] Crum L.A. (2015). Resource paper: Sonoluminescence. J. Acoust. Soc. Am..

[49] Adewuyi Y.G. (2001). Sonochemistry: Environmental science and engineering applications. Ind. Eng. Chem. Res..

[50] Pereira M.C., Oliveira L.C.A., Murad E. (2012). Iron oxide catalysts: Fenton and Fenton-like reactions—A review. Clay Miner..

[51] Didenko Y.T., Suslick K.S. (2002). The energy efficiency of formation of photons, radicals and ions during single-bubble cavitation. Nature.

[52] Gagol M., Przyjazny A., Boczkaj G. (2018). Wastewater treatment by means of advanced oxidation processes based on cavitation—A review. Chem. Eng. J..

[53] Rajoriya S., Bargole S., George S., Saharan V.K. (2018). Treatment of textile dyeing industry effluent using hydrodynamic cavitation in combination with advanced oxidation reagents. J. Hazard. Mater..

[54] Fang P., Tang Z., Chen X., Huang J., Chen D., Tang Z., Cen C. (2017). Split, partial oxidation and mixed absorption: A novel process for synergistic removal of multiple pollutants from simulated flue gas. Ind. Eng. Chem. Res..

[55] Li D., Xiao Z., Bin Aftab T., Xu S. (2018). Flue gas denitration by wet oxidation absorption methods: Current status and development. Environ. Eng. Sci..

[56] Lin F., Wang Z., Ma Q., He Y., Whiddon R., Zhu Y., Liu J. (2016). N_2_O_5_ Formation Mechanism during the Ozone-Based Low-Temperature Oxidation deNOx Process. Energy Fuels.

[57] Obvintseva L.A., Gubanova D.P. (2004). Determination of chlorine and chlorine dioxide in air with semiconductor sensors. J. Anal. Chem..

[58] Adewuyi Y.G., Sakyi N.Y. (2013). Simultaneous absorption and oxidation of nitric oxide and sulfur dioxide by aqueous solutions of sodium persulfate activated by temperature. Ind. Eng. Chem. Res..

[59] Yang C.-L., Shaw H. (1998). Aqueous absorption of nitric oxide induced by sodium chlorite oxidation in the presence of sulfur dioxide. Environ. Prog..

[60] Gong P., Li X. (2019). Promoting Effect of H^+^ and Other Factors on NO Removal by Using Acidic NaClO_2_ Solution. Energies.

[61] Park H.-W., Choi S., Park D.-W. (2015). Simultaneous treatment of NO and SO_2_ with aqueous NaClO_2_ solution in a wet scrubber combined with a plasma electrostatic precipitator. J. Hazard. Mater..

[62] Deshwal B.R., Jo H.-D., Lee H.-K. (2004). Reaction Kinetics of Decomposition of Acidic Sodium Chlorite. Can. J. Chem. Eng..

[63] Flagiello D., Di Natale F., Erto A., Lancia A. (2020). Wet oxidation scrubbing (WOS) for flue-gas desulphurization using sodium chlorite seawater solutions. Fuel.

[64] Flagiello D., Erto A., Lancia A., Di Natale F. (2022). Advanced Flue-Gas cleaning by wet oxidative scrubbing (WOS) using NaClO_2_ aqueous solutions. Chem. Eng. J..

[65] Chin T., Tam I.C.K., Yin C.-Y. (2022). Comparison of various chemical compounds for the removal of SO_2_ and NOx with wet scrubbing for marine diesel engines. Environ. Sci. Pollut. Res..

[66] Brogren C., Karlsson H.T., Bjerle I. (1998). Absorption of NO in an aqueous solution of NaClO_2_. Chem. Eng. Technol..

[67] Deshwal B.R., Jin D.S., Lee S.H., Moon S.H., Jung J.H., Lee H.K. (2008). Removal of NO from flue gas by aqueous chlorine-dioxide scrubbing solution in a lab-scale bubbling reactor. J. Hazard. Mater..

[68] Petkovšek M., Dular M. (2019). Cavitation dynamics in water at elevated temperatures and in liquid nitrogen at an ultrasonic horn tip. Ultrason. Sonochemistry.

[69] Brennen C.E. (2013). Cavitation and Bubble Dynamics.

[70] Hattori S., Taruya K., Kikuta K., Tomaru H. (2013). Cavitation erosion of silver plated coatings considering thermodynamic effect. Wear.

[71] Wei J., Luo Y., Yu P., Cai B., Tan H. (2009). Removal of NO from flue gas by wet scrubbing with NaClO_2_/(NH_2_)_2_CO solutions. J. Ind. Eng. Chem..

[72] Wang J., Zhong W. (2016). Simultaneous desulfurization and denitrification of sintering flue gas via composite absorbent. Chin. J. Chem. Eng..

[73] Zhao Y., Hao R., Qi M. (2015). Integrative process of preoxidation and absorption for simultaneous removal of SO2, NO and Hg0. Chem. Eng. J..

[74] Fang P., Tang Z., Chen X., Zhong P., Huang J., Tang Z., Cen C. (2018). Simultaneous removal of NOx and SO_2_ through a simple process using a composite absorbent. Sustainability.

[75] Mazzei A Injection Drawing of Model 287. https://mazzei.net/wp-content/uploads/2021/12/0287-REV-A-Inj_Drawing_2014-08-14_SECURED-1.pdf.

