# Supplementary material for: A Novel Method Based on Hydrodynamic Cavitation for Improving Nitric Oxide Removal Performance of NaClO2
Source: Int J Environ Res Public Health. 2023 Feb 19;20(4):3684. doi: 10.3390/ijerph20043684 (PMC9959747; doi:10.3390/ijerph20043684)
Supplement: Supplementary file 1 [file ijerph-20-03684-s001.zip › ijerph-2215427-supplementary.pdf]

Supplementary Information

A novel method based on hydrodynamic cavitation for improving nitric oxide removal performance of $NaClO_2$

Liguo Song [1, 2, †, *], Yuhang Wei [1, †], Chengqi Deng [1], Jingang Yang [1], Hao Sui [1], Feng Guo [1], Lingrun Meng [1], Xingda Zhao [1], Shiping Wei [1], Deping Sun [1], Zhitao Han [1,2], Minyi Xu [1,2], Xinxiang Pan [3]

Table S1. Factors affecting the NOx removal efficiency by composite NaClO2-containing oxidants.

Oxidant	Absorbent	Simulated gas concentration	Initial pH	Reaction temperature (°C)	Gas flow (L/min)	Oxidant concentration	Absorbent concentration	Maximum removal efficiency	Ref.
		NO (ppmv)							
$NaClO_2$	——	1000	6.00	25.0	1.5	$NaClO_2$ (40.00 mmol)	——	87.7% (NO)	[1]
$NaClO_2$	HA-NA	300	8.00	60.0	2.6	$NaClO_2$ (38.00 mmol)	HA-NA (20.00 mmol)	98.0% (NO)	[2]
$NaClO_2$	Na_2SO_3	500	10.00	25.0	0.5–1.3	$NaClO_2$ (80.00 mmol)	Na_2SO_3 (7.50 wt%)	92.0% (NO)	[3]
$NaClO_2$	NaOH	500	10.00	40.0	2.0	$NaClO_2$ (8.80 mmol)	NaOH (5.00 wt%)	82.0% (NO_x)	[4]
$NaClO_2$ + $(NH_2)_2CO$	——	1000	7.50	40.0	0.2	$NaClO_2$ (5.00 mmol) $(NH_2)_2CO$ (600.00 mmol)	——	98.0% (NO)	[5]
$NaClO_2$ + NaClO	——	500	5.50	50.0	1.0	NaClO (4.00 mmol) $NaClO_2$ (1.00 mmol)	——	85.0% (NO)	[6]
$NaClO_2$ + NaClO	——	350	6.00	55.0	0.5	NaClO (3.00 mmol) $NaClO_2$ (4.00 mmol)	——	90.8% (NO)	[7]
$NaClO_2$ + H_2O_2	$Ca(OH)_2$	500	4.50	150.0	3.0	$NaClO_2$ (100.00 mmol) H_2O_2 (4.00 mmol)	$Ca(OH)_2$ (140.00 mmol)	85.5% (NO)	[8]
$NaClO_2$ + $Na_2S_2O_8$	HA-NA	407	9.00	140.0	3.0	$NaClO_2$ (4.00 wt%) $Na_2S_2O_8$ (4.00 wt%)	HA-NA (20.00 mmol)	82.7% (NO)	[9]

$NaClO_2$ + NaBr	$Ca(OH)_2$	550	7.00	140.0	3.0	$NaClO_2$ (1.50×10^3 mmol) NaBr (50.00 mmol)	$Ca(OH)_2$ (140.00 mmol)	91.0% (NO)	[10]
$NaClO_2$ + $Na_2S_2O_8$	NaOH	500	12.00	50.0	2.0	$NaClO_2$ (200.00 mmol) $Na_2S_2O_8$ (200.00 mmol)	NaOH (1.25×10^3 mmol)	91.1% (NO_x)	[11]
$NaClO_2$	——	1000	3.50	45.0	1.0	$NaClO_2$ (1.00 mmol)	——	100% (NO_x)	This paper

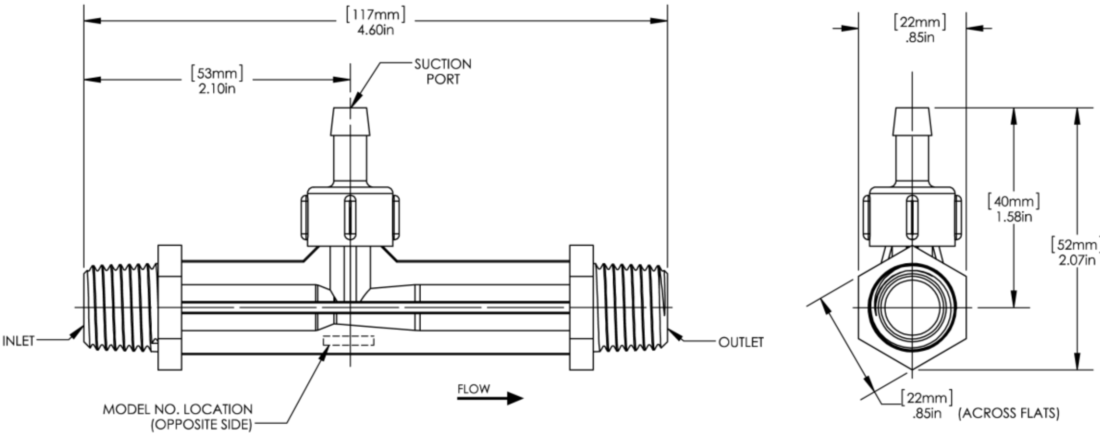

Figure S1. The structure and dimensions of HC reactor from Mazzei [12]

References

1. Han, Z.; Lan, T.; Han, Z.; Yang, S.; Dong, J.M.; Sun, D.D.; Yan, Z.; Pan, X.; Song, L. Simultaneous removal of NO and SO_2 from exhaust gas by cyclic scrubbing and online supplementing pH-buffered $NaClO_2$ solution. *Energy Fuels* **2019**, *33*, 6591-6599, doi:10.1021/acs.energyfuels.9b01106.
2. Hao, R.; Zhang, Y.; Wang, Z.; Li, Y.; Yuan, B.; Mao, X.; Zhao, Y. An advanced wet method for simultaneous removal of SO2 and NO from coal-fired flue gas by utilizing a complex absorbent. *Chem. Eng. J.* **2017**, *307*, 562-571, doi:10.1016/j.cej.2016.08.103.
3. Liu, Z.-H.; Xu, H.-Z.; Li, Y.-B.; Luo, Y.; Zhang, L.-L.; Chu, G.-W. Nox removal from gas mixture intensified by rotating packed bed with $NaClO_2$ preoxidation. *Chem. Eng. J.* **2022**, *430*, 132671, doi:10.1016/j.cej.2021.132671.
4. Fang, P.; Tang, Z.; Chen, X.; Huang, J.; Chen, D.; Tang, Z.; Cen, C. Split, partial oxidation and mixed absorption: A novel process for synergistic removal of multiple pollutants from simulated flue gas. *Ind. Eng. Chem. Res.* **2017**, *56*, 5116-5126, doi:10.1021/acs.iecr.6b05029.
5. Wei, J.; Luo, Y.; Yu, P.; Cai, B.; Tan, H. Removal of NO from flue gas by wet scrubbing with $NaClO_2/(NH_2)_2CO$ solutions. *J. Ind. Eng. Chem.* **2009**, *15*, 16-22, doi:10.1016/j.jiec.2008.07.010.
6. Zhao, Y.; Guo, T.-X.; Chen, Z.-Y.; Du, Y.-R. Simultaneous removal of SO_2 and NO using M/$NaClO_2$ complex absorbent. *Chem. Eng. J.* **2010**, *160*, 42-47, doi:10.1016/j.cej.2010.02.060.
7. Wang, J.; Zhong, W. Simultaneous desulfurization and denitrification of sintering flue gas via composite absorbent. *Chin. J. Chem. Eng.* **2016**, *24*, 1104-1111, doi:10.1016/j.cjche.2016.04.005.
8. Zhao, Y.; Hao, R.; Yuan, B.; Jiang, J. Simultaneous removal of SO_2, NO and Hg^0 through an integrative process utilizing a cost-effective complex oxidant. *J. Hazard. Mater.* **2016**, *301*, 74-83, doi:10.1016/j.jhazmat.2015.08.049.
9. Hao, R.; Yang, S.; Yuan, B.; Zhao, Y. Simultaneous desulfurization and denitrification through an integrative process utilizing $NaClO_2/Na_2S_2O_8$. *Fuel Process. Technol.* **2017**, *159*, 145-152, doi:10.1016/j.fuproc.2017.01.018.
10. Zhao, Y.; Hao, R.; Qi, M. Integrative process of preoxidation and absorption for simultaneous removal of SO2, NO and Hg0. *Chem. Eng. J.* **2015**, *269*, 159-167, doi:10.1016/j.cej.2015.01.064.
11. Fang, P.; Tang, Z.; Chen, X.; Zhong, P.; Huang, J.; Tang, Z.; Cen, C. Simultaneous removal of NOx and SO_2 through a simple process using a composite absorbent. *Sustainability* **2018**, *10*, 4350, doi:10.3390/su10124350.
12. Mazzei. A Injection Drawing of model 287. Available online: https://mazzei.net/wp-content/uploads/2021/12/0287-REV-A-Inj_Drawing_2014-08-14_SECURED-1.pdf (accessed on
